# Better clinical outcome of total knee arthroplasty for rheumatoid arthritis with perioperative glucocorticoids and disease-modifying anti-rheumatic drugs after an average of 11.4-year follow-up

**DOI:** 10.1186/s13018-021-02232-9

**Published:** 2021-01-27

**Authors:** Yi Ren, Qi Yang, Tim Luo, Jin Lin, Jin Jin, Wenwei Qian, Xisheng Weng, Bin Feng

**Affiliations:** 1 Department of Orthopedic Surgery, Peking Union Medical College Hospital, Peking Union Medical College, Beijing, 100730 China; 2 Department of Orthopedics, First Hospital of Harbin, Harbin, China; 3 Doctor of Medicine Program, University of Alberta, Edmonton, AB Canada

**Keywords:** Total knee arthroplasty, Rheumatoid arthritis, Disease-modifying anti-rheumatic drugs, Glucocorticoids, Clinical outcome, Complication

## Abstract

**Background:**

Previous evidence suggested that perioperative anti-rheumatic therapy for patients receiving total knee arthroplasty (TKA) helped improve postoperative rehabilitation for rheumatoid arthritis (RA), yet long-term effects and outcomes of perioperative drug therapy in TKA presently remain unclear. This study investigated whether perioperative treatment with glucocorticoids (GC) and disease-modifying anti-rheumatic drugs (DMARDs) can improve clinical outcomes for patients with RA undergoing TKA.

**Methods:**

Patients between January 2000 and December 2011 were allocated into three groups based on perioperative drug therapy: A, control group (no GC or DMARDs), B, DMARD group (DMARDs given without GC), and C, co-therapy group (DMARDs plus GC). The patients were followed up for average 11.4 years. Baseline characteristics, pre- and post-operative Hospital for Special Surgery score (HSS), laboratory parameters, and complications were recorded by follow-up.

**Results:**

Fifty-six RA patients undergoing 91 TKAs were included in this study. Patients who received perioperative GC with DMARDs (group C) achieved larger/increased range of motion (ROM) (C:122.17 vs A:108.31 vs B:108.07, *p* = 0.001, partial eta squared (*η*^2^ p) = 0.18) at 1 year, better HSS score (C, 83.01 vs A, 79.23 vs B, 77.35, *p* = 0.049, *η*^2^ p = 0.067), pain relief (C, 1.09 vs A, 1.17 vs B, 1.75, *p* = 0.02, *η*^2^ p = 0.094), and ROM (C, 130.81 vs A, 112.82 vs B, 113.58, *p* = 0.001, *η*^2^ *p* = 0.142) at latest follow-up comparing with the other treatment groups. No differences were noted in laboratory tests, blood loss, volume of transfusion, or complications among groups.

**Conclusions:**

Compared with the other perioperative anti-rheumatic treatments, the combination of GC and DMARDs results in improved HSS score, better function, larger range of motion, and reduced postoperative pain for TKA patients with RA in the long term. Further investigation is warranted to look for a better understanding of more specific medication effects and strike a good balance between the benefits and complications for long-term pharmacotherapy.

## Introduction

Rheumatoid arthritis (RA) is often characterized as an inflammatory autoimmune disease, causing cartilage and bone damage with progression to joint malformation and eventual loss of function. Knee lesions are commonly seen in chronic RA patients, gradually impairing ambulatory capacity and subsequent quality of life [1–3].

For end-stage knee arthropathy of RA patients, total knee arthroplasty (TKA) is an effective approach to achieve outstanding restoration of knee function. However, it is still an open question how anti-rheumatic medication management should be designed perioperatively. Orthopedic surgeons should weigh the balance between risk of infection and flare during arthroplasty surgery. According to conventional practice and international consensus, it is suggested that the continuation of anti-rheumatic drug therapy (except for biologic agents) helps control disease activity and improve postoperative rehabilitation [4]. Previous literature reported that perioperative use of glucocorticoids (GC) and disease-modifying anti-rheumatic drugs (DMARDs) contribute to a lower disease activity level in 1 year after surgery, avoiding exacerbation of the disease, measured by Disease Activity Score including 28-joint count (DAS28) [5]. However, clinical data of long-term effects of perioperative drug therapy in TKA presently remain sparse. This study intends to evaluate short-term and long-term clinical outcome and postoperative complications associated with preoperative GC and DMARD use in RA patients undergoing TKA surgery.

## Materials and methods

This is a retrospective observational study designed under the Strengthening the Reporting of Observational Studies in Epidemiology (STROBE) guidelines and was approved by the institutional review board [6] (number of approval: S-K1025). Informed written consents were obtained from patients for publication.

### Patient selection and management

**Fig. 1:**
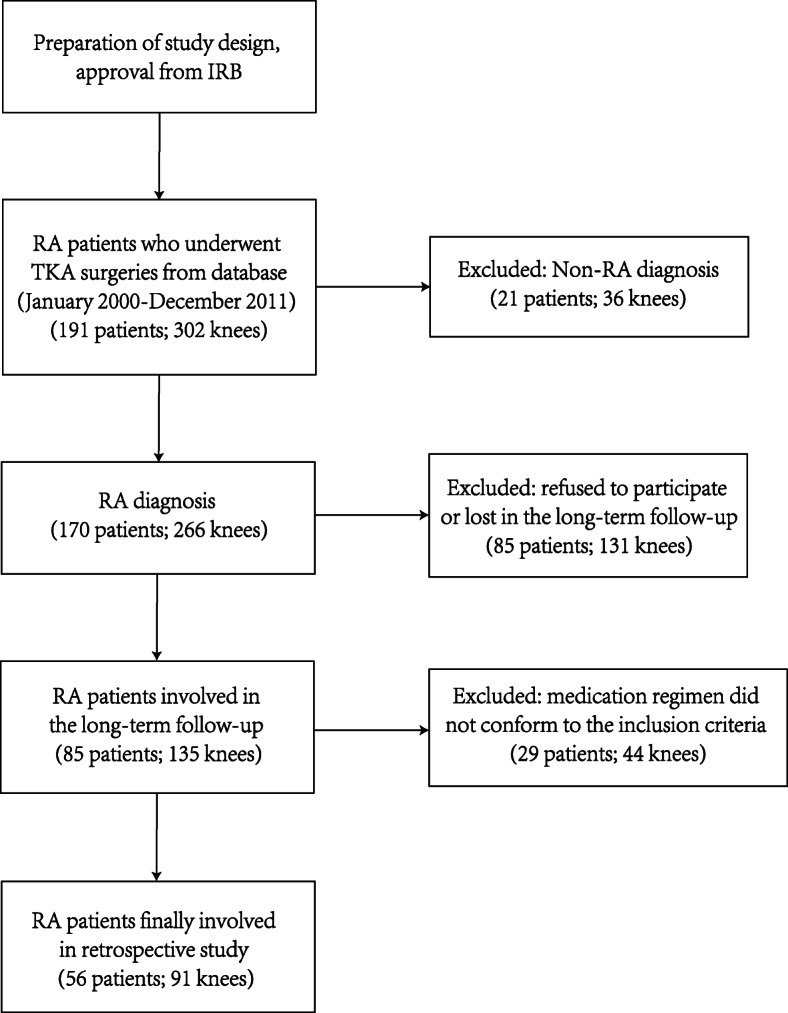
Flow chart of RA patient screening process. IRB, institutional review board; RA, rheumatoid arthritis; TKA, total knee arthroplasty

The process of patient selection is shown in flow chart (Fig. 1). To reach a long-term follow-up result, patients included in our study should (1) be diagnosed with RA undergoing TKAs between January 2000 and December 2011; (2) receive treatment with DMARDs or DMARDs plus GC in curative dose for a minimum of 1 year continually after surgery, or just without anti-rheumatic medication; (3) agree to participate in our study and follow-up. Exclusion criteria included (1) diagnosis with other joint diseases (i.e. osteoarthritis, ankylosing spondylosis); (2) disobedience to receive anti-rheumatic medication treatment continually in the 1 year postoperatively; (3) patients who declined to be followed up or could not provide complete data.

RA disease status was verified in all patients through history of rheumatologist diagnosis according to standardized diagnostic criteria before admission. The enrolled patients underwent cemented TKA with posterior stabilized prosthesis through medial parapatellar approach. Surgical techniques and the type of prostheses were decided by attending surgeons. Patients received thromboprophylaxis with subcutaneous injection of low-weighted molecular heparin (nadroparin calcium, 0.4 ml, q.d.; or enoxaparin sodium, 0.4 ml, q.d.) in a total of 7–14 days depending on their walking ability and coagulation function after surgery. Sequential compression devices began within 24 h of operation for thromboembolic prophylaxis and continued during the hospitalization period.

Based on perioperative anti-rheumatic medication therapy, patients were organized into three treatment groups:

A. Control group (no anti-rheumatic drugs used)
B. DMARD group (conventional or biologic DMARD use with no GC)
C. Co-therapy group (DMARD and GC use)

In group A, the disease was treated and controlled with immunosuppressants before. The immunosuppressants were stopped after consultation with their attending physicians. Therefore, no further anti-rheumatic drugs were used during the perioperative period. Non-steroid anti-inflammatory drugs (NSAIDs) were administered if necessary. Follow-up was then conducted through outpatient clinic questionnaires or via telephone. The patients were regularly followed at postoperative 3 months, 1 year, and annually thereafter. In summary, the follow-up of the patients ranged from 7–16 years after surgery, with an average of 11.4 years.

In our study, no patients received GC monotherapy. Conventional DMARDs treatment was continued during surgery for groups B and C; however, all biologic DMARDs were stopped 4 weeks before surgery and restarted at least 1 week postoperatively depending on medication, wound healing, and disease status. Oral NSAIDs were given in each group as needed to control acute pain.

### Data extraction and collection

Data in our study came from our database and the outpatient-clinic follow-up. Information on patient demographics included age, gender, body mass index (BMI), information regarding NSAIDs, GC and DMARD therapy, the type of prothesis for each operated knee and whether unilateral or bilateral TKA was performed (Tables 1 and 2).

**Table 1:** Patient demographics

	Group A	Group B	Group C	*p* value
Number of patients (*n*)	20	15	21	/
Number of knees (*n*)	29	26	36	/
Number of operated knees (unilateral/bilateral) (*n*)	11/9	4/11	6/15	/
Age (years)	53.91 ± 11.02	53.51 ± 11.11	48.21 ± 11.94	0.88
BMI (kg/m^2^)	23.42 ± 2.86	22.52 ± 3.84	22.12 ± 4.24	0.40
Sex (female/male) (*n*)	14/6	13/2	18/3	0.35
DAS28	2.56 ± 0.63	2.68 ± 0.43	2.36 ± 0.63	0.09
Flexion deformity (%)	15 (51.72)	18 (69.23)	22 (61.11)	0.41
General anesthesia/non general anesthesia(n)	12/8	12/3	13/8	0.31
Duration of medication treatment after surgery(years)	N/A	8.13 ± 2.42	8.33 ± 2.63	0.80
Pre-op HSS	43.61 ± 16.32	42.02 ± 14.32	42.01 ± 17.73	0.91
Pre-op ROM (°)	78.51 ± 34.79	78.73 ± 30.82	89.81 ± 28.22	0.25
Pre-op pain	4.96 ± 0.96	4.65 ± 1.02	3.48 ± 0.87	0.06
Pre-op function	18.28 ± 7.11	18.85 ± 7.79	18.00 ± 9.09	0.92
Pre-op VAS	5.93 ± 0.59	6.37 ± 0.63	6.76 ± 0.53	0.12
Pre-op WBC (*10^9^/L)	6.75 ± 1.81	7 ± 1.92	7.05 ± 2.58	0.88
Pre-op HGB (g/L)	119.12 ± 17.09	111.21 ± 14.22	116.22 ± 18.21	0.34
Pre-op CRP (mg/L) #	4.5 (1.6–15.3)	15.0 (4.0–27.9)	8.5 (1.7–25.9)	0.18
Pre-op ESR (mm/h) #	27.0 (12.0–64.0)	58.0 (38.5–78.0)	32.0 (14.5–65.3)	0.06
Pre-op RF (U/ml) #	39.0 (10.4–106.0)	64.0 (28.0–189.5)	31.2 (10.7–131.3)	0.32

*n* case number, *TKA* total knee arthroplasty, *BMI* body mass index, *DAS28* Disease Activity Score 28-joint, *Pre-op* preoperative, *HSS* Hospital for Special Surgery score, *ROM* range of motion, *VAS* visual analog scale, *WBC* white blood cell, *HGB* hemoglobin, *CRP* C-reaction protein, *ESR* erythrocyte sedimentation rate, *RF* rheumatoid factor, *N/A* not applicable

^#^ Data were described as “median (interquartile range)” with Kruskal-Wallis test

**Table 2:** Perioperative medications in groups B and C

	No. of cases	Median dosage	Median treatment duration
Group B			
LEF	5	15 mg daily	8 years
MTX	9	12.5 mg weekly	10 years
SASP	1	2000 mg daily	5 years
TGP	1	1200 mg daily	0.5 years
TG	7	40 mg daily	14 years
ETN	2	25 mg biweekly	0.5 years
Group C			
IGU	2	50 mg daily	1 years
MTX	18	12.5 mg weekly	12 years
PA	1	1000 mg daily	6 years
SASP	1	2000 mg daily	4 years
TG	13	60 mg daily	8 years
ETN	3	25 mg biweekly	0.5 years
PRDL	4	7.5 mg daily	11.5 years
PRED	16	10 mg daily	12 years
MPS	1	8 mg bi-daily	21 years

*LEF* leflunomide, *MTX* methotrexate, *TGP* total glucosides of paeony, *TG* tripterygium glycosides, *SASP* salicylazosulfapyridine, *ETN* etanercept, *IGU* iguratimod, *PA* penicillamine, *PRDL* prednisolone, *PRED* prednisone, *MPS* methylprednisolone

For clinical evaluation, Hospital of Special Surgery (HSS) knee score, ROM were recorded preoperatively, at the time of 1 year and latest follow-up. The pain was further assessed using a visual analog scale (VAS). Preoperative DAS28 was also recorded. Laboratory test involved white blood cells (WBC), hemoglobin (HGB), C reaction protein (CRP), erythrocyte sedimentation rate (ESR), and rheumatoid factor (RF). The volume of wound drainage, need for postoperative blood transfusion, the postoperative temperature at days 1 and 3 (T pod 1 and 3) were involved for analysis. The total perioperative blood loss was calculated based on the “hemoglobin balance” theory [7].

Short-term complications within 3 months postoperatively, such as acute infection, delayed wound healing, and RA flare, were recorded and were categorized into systematic, wound, and surgical issues. Long-term complications related to the index operation were also recorded/noted down, such as periprosthetic joint infection (PJI), fracture, prosthesis loosening, and need for surgical revision.

### Statistical analysis

Statistical analysis was performed using IBM SPSS Statistics software version 25 (IBM Corporation, Armonk, USA). Continuous data with normal distribution were reported as mean and standard deviation (SD), while non-normally distributed data were presented as median and interquartile range. Analysis of variance (ANOVA) was used to compare quantitative data among the three treatment groups with subsequent Bonferroni pairwise comparisons. Kruskal-Wallis test was indicated for non-normally distributed data. Considering that disease activity might affect the value of pain evaluation, HSS scoring, and ROM, we adopted DAS28 as a covariate of these results and used analysis of covariance method. Besides, partial eta squared (*η*^2^ p) was employed for calculating effect size with the following cut-off values to interpret 0.01 to 0.06 as small effect, 0.06 to 0.14 as medium effect, and above 0.14 as large effect. Chi-square test and Fisher’s exact test were used to analyze qualitative variables. In Fisher’s exact test, Bonferroni correction was used for adjusting the significance limit to *P* < 0.017, while significance was defined as *P* < 0.05 for other tests.

## Results

### Demographic and disease features

This study included 91 TKA operations performed on 56 RA patients in our hospital, with a mean patient age of 51.7 years. The mean duration of RA medication treatment after surgery was approximately 8 years for both groups B and C. No statistically significant difference was seen between any treatment groups in patient demographics, preoperative or postoperative laboratory tests, estimated blood loss, and temperature at POD1 (Tables 1 and 3). There was no significant difference of disease activity status for the three groups after evaluation with DAS28.

**Table 3:** Postoperative clinical outcomes and clinical outcomes during perioperative period, at the 1-year and latest follow-up

	Group A	Group B	Group C	*p*	A vs B	A vs C	B vs C
Perioperative period							
Drainage (ml)#	500.0(320.0–640.0)	430.0(330.0–730.0)	540.0(265.0–833.5)	0.98	/	/	/
Blood transfusion(ml) #	400.0(0–600.0)	600.0(300.0–800.0)	600.0(400.0–800.0)	0.16	/	/	/
Post-op WBC (*10^9^/L)	10.63 ± 3.72	11.87 ± 3.81	11.91 ± 3.2	0.34	0.68	0.60	0.99
Post-op HGB (g/L)	106.57 ± 5.40	97.59 ± 6.31	98.59 ± 5.48	0.06	0.11	0.13	1.00
HGB drop (g/L)	17.83 ± 14.51	14.91 ± 11.16	24.39 ± 18.23	0.13	0.99	0.44	0.17
Estimated blood loss (ml)#	762.96 (359.29–1282.42)	743.58 (524.72–1341.36)	818.95 (601.09–1104.66)	0.72	/	/	/
T pod 1 (°C)	37.94 ± 0.39	37.82 ± 0.64	37.59 ± 0.72	0.20	0.99	0.23	0.99
T pod 3 (°C)	37.52 ± 0.29	37.21 ± 0.63	37.09 ± 0.51	0.03	0.48	0.02**	0.85
Post-op ROM before discharge (°)	100.21 ± 5.86	108.43 ± 6.26	108.05 ± 5.33	0.08	0.17	0.15	1.00
At the 1-year follow-up							
ROM (°)	108.31 ± 5.37	108.07 ± 5.75	122.17 ± 4.89	0.001	1.00	0.001**	0.001***
VAS	2.24 ± 0.68	2.71 ± 0.76	2.24 ± 0.73	0.34	0.48	1.00	0.42
HSS	72.22 ± 2.36	70.91 ± 2.52	74.88 ± 2.14	0.06	1.00	0.30	0.06
HSS pain score	22.79 ± 2.43	22.43 ± 2.56	23.23 ± 2.01	0.78	1.00	1.00	1.00
HSS function score	13.72 ± 1.43	12.84 ± 1.53	14.34 ± 1.30	0.34	1.00	1.00	0.43
At the latest follow-up							
ROM (°)	112.82 ± 7.82	113.58 ± 8.36	130.81 ± 7.12	0.001	1.00	0.003**	0.008***
VAS	1.17 ± 0.43	1.75 ± 0.52	1.09 ± 0.39	0.02	0.10	1.00	0.04***
HSS	79.23 ± 3.31	77.35 ± 3.53	83.01 ± 3.01	0.049	1.00	0.27	0.045***
HSS pain score	25.67 ± 2.37	24.52 ± 2.68	26.75 ± 2.47	0.15	0.82	0.73	0.12
HSS function score	14.60 ± 1.66	13.71 ± 1.77	15.56 ± 1.51	0.30	1.00	1.00	0.36

*Post-op* postoperative, *HSS* Hospital for Special Surgery, *ROM* range of motion, *VAS* visual analog scale, *WBC* white blood cell, *HGB* hemoglobin, *T pod* 1/T pod 3, temperature in postoperative days 1 and 3

*Difference between groups A and B is significant

** Difference between groups A and C is significant

*** Difference between groups B and C is significant

^#^Data were described as “median (interquartile range)” with Kruskal-Wallis test

### Type, dose, and duration of anti-rheumatic medication

The patients accepted non-selective (Diclofenac Sodium, Ibuprofen) or selective Cox-2 inhibitor (celecoxib) treatment for pain control when necessary. No statistical difference was observed in terms of the proportion of non-selective or selective NSAIDs use (*P* = 0.82). In group B, the 15 patients were treated with conventional DMARDs as a single drug regimen. Eight of them (53.3%) accepted treatment with total glucosides of paeony (TGP) or tripterygium glycosides (TG), which is known as anti-rheumatic drugs extracted from plants. Two patients in group B (13.3%) were given a mix of conventional and biologic DMARD. All patients in group C received GC plus DMARDs, among whom 20 patients (95.2%) received conventional DMARD monotherapy and 13 of the 20 patients received TGP. One patient (4.8%) received the combination therapy with two conventional DMARDs, and three patients (14.3%) received a mix of biologic and conventional DMARD regimen. No patients received GC intra-articular injections. (Table 2)

### Outcome measurements

Statistical analysis showed no significant difference among patients in three groups in terms of VAS (*P* = 0.34) and HSS pain score (*P* = 0.78) at the 1-year follow-up. At the latest follow-up, the patients in group C showed significant improvement in VAS score compared with those in group B (*P* = 0.04, *η*^2^ p = 0.094), but no statistical difference in HSS pain score (*P* = 0.12). There was no statistical difference when comparing group A with group B or C in terms of VAS and HSS pain score (Table 3).

At the postoperative 1-year follow-up, patients in group C had an increased degree of ROM than the other two groups (*p* = 0.001 and 0.001, *η*^2^ p = 0.18). At the latest follow-up, patients in group C also had the significantly greater improvement of ROM compared with both groups A and B (*p* = 0.003 and *p* = 0.008 respectively, *η*^2^ p = 0.142), while there was no difference of ROM between groups A and B at the latest follow-up (*p* = 1.00) (Table 3).

**Fig. 2:**
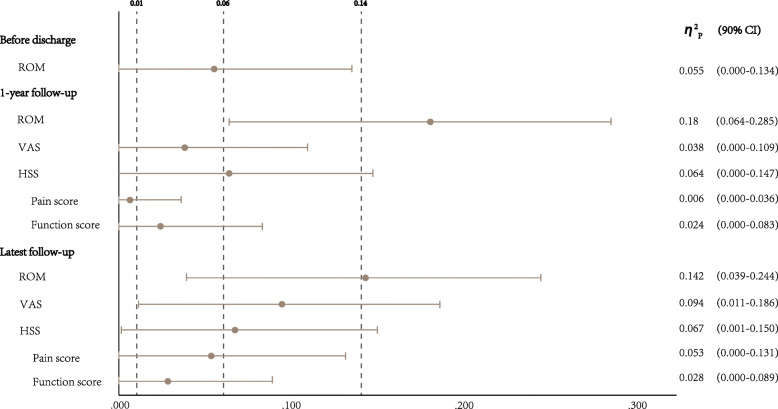
Effect size (described with η^2^_p_ and 90% CI) of the clinical results. The vertical dotted lines labeled with number 0.01, 0.06, and 0.14 indicated the boundary of small, medium, and large effect, respectively. *η2p* partial eta squared, *CI* confidence interval, *ROM* range of motion, *HSS* Hospital for Special Surgery, *VAS* visual analog scale

There was no statistical significance of HSS knee score among three groups at the postoperative 1 year (*p* = 0.06) (Table 3). At the latest follow-up, patients in group C had significantly higher HSS score than patients in group B (*p* = 0.045, *η*^2^ p = 0.067) (Table 3), while there was no significant difference of HSS score between group A and B (*p* = 1.00) as well as groups A and C (*p* = 0.27) (Table 3 and Fig. 2).

### Postoperative complication

The details of complications were shown in Table 4. For short-term complication, there was no complication of DVT in group C, while one in group A and four in group B. The DVT ratio in group C was lower than that in group B, and pairwise comparison showed no statistical significance between the two groups (*p* = 0.03). As for other short-term complication, there was no difference of systematic, wound, and surgical complication among the three groups.

**Table 4:** Complications during follow-up

	Group A	Group B	Group C	P	A vs B	A vs C	B vs C
Deep venous thrombosis (cases)	1	4	0	0.03	0.14	1.00	0.03
Number of other short-term complications§	4†	0	4 ‡	0.15	/	/	/
Systematic	2	0	3	0.36	/	/	/
Wound	1	0	0	0.99	/	/	/
Surgical	1	0	1	0.99	/	/	/
Long term	0	0	1	0.63			

†Included allergic shock after blood transfusion; hemarthrosis; subcutaneous adiponecrosis and common peroneal nerve compression; sciatic nerve injury

‡Included urinary infection; herpes zoster; compromised sensory function of planta pedis and plantar flexion ability of the first toe; thrombocytopenia

§ Short-term complications are defined as those no more than 3 months after surgery

After average 11.4 years follow-up, no aseptic loosening, instability, or periprosthetic fracture was reported in our study. One case developed prosthetic joint infection (PJI) in group C at 3 years after index surgery and underwent two-stage revision surgery.

## Discussion

Rheumatoid arthritis is an inflammatory autoimmune disease causing progressive articular destruction and malformation. GC and DMARD managements are routinely used to control chronic RA disease activity. Anti-inflammatory glucocorticoids are commonly used to reduce pain, stiffness, and to slow progressive bone erosion [3, 8, 9]. DMARDs is another cornerstone class of RA medication, consisting of conventional and biologic DMARDs, which slows disease progression by targeting and resolving inflammatory disease pathophysiology. The combination of GC and DMARDs provides additive benefit and is reported to reduce the risk for joint replacement and radiographic disease progression compared with drug monotherapy [4, 10, 11]. TKA remains the definitive treatment in end-stage disease with severe malformation, which can restore joint function [12, 13].

Inflammatory control and suppression of RA disease activity are critical to TKA perioperative management according to the American College of Rheumatology/American Association of Hip and Knee Surgeons 2017 Guideline. When RA patients underwent elective TKA, it was recommended to continue the current dose of conventional DMARDs, but to withhold all current biologic agents prior to surgery. Once the wound shows evidence of healing and no non-surgical site infections occurs, the biologic therapy should be resumed [14]. In this study, we aimed to observe the long-term clinical outcome of perioperative anti-rheumatic medication use in TKA surgery.

It was reported that the intervention of anti-rheumatic drugs could improve the short-term clinical outcome and patient satisfaction [15]. Results of the present study also showed that perioperative treatment with DMARDs and GC co-therapy could result in significant long-term improvement in HSS score, VAS score, and patient satisfaction compared with patients managed with only DMARDs, and corresponding effect sizes were characterized from medium to large effect. The possible reason was that both GC and DMARDs could exert synergetic effect to lower perioperative disease activity. Goodman et al. reviewed the availability and safety of anti-rheumatic medications in perioperative treatment [16]. Systematic GC use was regarded effective to accelerate recovery, functional rehabilitation after arthroplasty surgery among RA patients through its pain-relieving and anti-emetic effect shortly after surgery [1, 13]. On the other side, administration of DMARDs can mitigate soft tissue damage, reduce inflammation, and therefore improve joint symptoms by interfering with complex immune pathways in the pathogenesis of RA, especially in the long run [17–19]. Therefore, the combination of these two kinds is conducive to functional improvement from the short to long term.

We also found ROM significantly increased in co-therapy group than either DMARDs-only or no anti-rheumatics groups in short- and long-term follow-up. These results agree with previous literature supporting treatment with GC combined with DMARDs to achieve better patient satisfaction and prognosis [1, 13, 17–20]. We found no significant difference in HSS score, VAS pain, or ROM was observed between patients given DMARD alone and the control group. Possible reason might be that pain from other joints damaged by systemic RA may interfere with the assessment of knee joints after TKA surgery, without the rapid onset of GC analgesic effects [21, 22]. Furthermore, the NSAIDs used in group B could also inhibit inflammation shortly after surgery, so did GC. Overall, this study supports the co-therapy of perioperative DMARD and GC to improve knee function, patient satisfaction, and decrease pain.

We also reported DVT events in our three cohorts, with one case in the control, four in the DMARD therapy group, and no cases in the GC and DMARDs co-therapy group. DVT is a common complication after TKA procedure. With the use of GC, the morbidity rate increases, mainly due to an induced hypercoagulable state [23]. White et al. reported perioperative GC therapy for patients undergoing spinal surgery had an increased risk of DVT than those without GC therapy [23]. Continuing systematic use of GC rendered higher risk of venous thromboembolism with an approximate 2-fold increase [24]. But the impact of GC use for patients receiving TKA still remained unclear. The result of study might indicate no increase in DVT risk associated with co-therapy use among RA patients in the long term. Nevertheless, risk evaluation should be performed carefully for individuals before decision.

Although our study shows GC and DMARDs to improve joint function, mobility, and patient satisfaction with limited complication, RA patients are still at particular risk for infection, either systemic or local [8]. Prosthetic joint infection (PJI) is a form of local infection, occurs in about 0.5–2% of TKAs and is a disastrous surgical complication after TKA [4, 25, 26]. A meta-analysis describes increased risk for PJI up to 3 years postoperatively in patients using continuous GC therapy [25]. There is also a correlation between the intra-articular injection of GC and PJI after knee arthroplasty [27]. Nevertheless, perioperative inflammatory damage of RA disease can potentially cause periarticular bone degradation and implant loosening [2, 28, 29]. Studies have also suggested that a standard dose of GC perioperatively can help with inflammation without drastically increasing infectious risk during the surgical period [30]. Only one case of PJI developed at 3 years follow-up in our entire study, and occurred in the GC and DMARD co-therapy group. According to our study, it is very difficult to draw any sound conclusion about the GC treatment for RA patients and the development of PJI.

Limitations are inevitable in all studies including this one. First, the study cohort was relatively small, as all patient profiles were acquired from the database of a single medical center. Although we were able to draw conclusions from our study, and patients we selected were over 11.4 years follow-up which have covered the breadth of conventional RA presentations, a larger population may provide greater statistical efficacy and may permit a more comprehensive analysis of surgical complications. Second, many different types of conventional and biologic DMARDs were analyzed in combination, which potentially may mask specific medication affects. However, more detailed categorizing and analysis of patient drug regimens would have required a much larger study population and combined analysis of DMARDs allow our study results to be better reflected and applied to a heterogeneous general RA population. Third, approximately half of our patients contributed two knees in our study. Even though we separately analyzed under several outcomes such as HSS and ROM, lack of the interaction data between two knees might lead to the loss of statistical power and an increase in potential biases when interpreting results. Fourth, we did not evaluate the side-effect of long-term use of GC treatment, such as osteoporosis, and gastric-intestinal side-effect. Future study is needed to answer this concern. The clinicians should balance the benefit and the complication for long-term GC treatment in practice. Finally, our long-term analysis period with an average 11.4-year follow-up inevitably contributed to cohort attrition due to death or an inability or refusal to follow-up, which further limited our study size. And the medical information we obtain from their close relatives is rarely as accurate as the patient’s self-reported history.

In conclusion, this study suggests that perioperative co-pharmacotherapy with GC and DMARDs, compared with the other treatments, can better improve long-term TKA clinical outcomes and knee recovery measured through HSS knee score, joint ROM, and VAS pain, while does not increase the surgical-related complications. Further investigation is warranted with a larger cohort size to have a better understanding of more specific medication effects and strike a good balance between the benefits and complications for long-term co-pharmacotherapy.

## Data Availability

The datasets generated and/or analyzed during the current study are not publicly available due to the local policy but are available from the corresponding author on reasonable request.

## Abbreviations

TKA: Total knee arthroplasty
RA: Rheumatoid arthritis
GC: Glucocorticoids
DMARD: Disease-modifying anti-rheumatic drug
ROM: Range of motion
HSS: Hospital for Special Surgery
STROBE: Strengthening the Reporting of Observational Studies in Epidemiology
VAS: Visual analog scale
DAS-28: Disease Activity Score including 28-joint count
WBC: White blood cells
HGB: Hemoglobin
CRP: C-reaction protein
ESR: Erythrocyte sedimentation rate
RF: Rheumatoid factor
DVT: Deep vein thrombosis
POD: Postoperative day
